# Supplementary material for: Multidimensional analysis of Gammaherpesvirus RNA expression reveals unexpected heterogeneity of gene expression
Source: PLoS Pathog. 2019 Jun 5;15(6):e1007849. doi: 10.1371/journal.ppat.1007849 (PMC6576797; doi:10.1371/journal.ppat.1007849)
Supplement: S1 Text — Viral and host probes for (Table A) PrimeFlow analysis, (Table B) TaqMan PCR, and (Table C) qPCR. (PDF) [file ppat.1007849.s001.pdf]

Supplemental Text 1 for "Multidimensional analysis of Gammaherpesvirus RNA expression reveals unexpected heterogeneity of gene expression"

Lauren M. Oko[1], Abigail K. Kimball[2], Rachael E. Kaspar[2], Ashley N. Knox[1], Carrie B. Coleman[1], Rosemary Rochford[1], Tim Chang[3], Benjamin Alderete[4], Linda F. van Dyk[1]*, Eric T. Clambey[2]*#

Affiliations:

[1] Department of Immunology and Microbiology, University of Colorado Denver | Anschutz Medical Campus, Aurora, CO, 80045, USA

[2] Department of Anesthesiology, University of Colorado Denver | Anschutz Medical Campus, Aurora, CO, 80045, USA

[3] MilliporeSigma, a business of Merck KGaA, Darmstadt, Germany (Seattle, WA, USA)

[4] Luminex Corporation, Austin, TX, USA

*** Co-Corresponding authors:**

Eric T. Clambey, eric.clambey@ucdenver.edu, 303-724-7783 (phone)

Linda F. van Dyk, linda.vandyk@ucdenver.edu, 303-724-4207 (phone)

[#] **Lead author** for MS correspondence

Table A: Viral and host probes for PrimeFlow analysis

Target and probe name	Fluorochrome	ThermoFisher Probe number	Target species
Murid Herpesvirus ORF18 Type 4	Alexa Fluor 488	VF4-6000512	γHV68
Murid Herpesvirus ORF18 Type 6	Alexa Fluor 750	VF6-6001307	γHV68
Murid Herpesvirus ORF64 Type 10	Alexa Fluor 568	VF10-6001306	γHV68
Murid Herpesvirus ORF72 Type 1	Alexa Fluor 647	VF1-20941	γHV68
Murid Herpesvirus ORF73 Type 1	Alexa Fluor 647	VF1-17077	γHV68
Murid Herpesvirus TMER Type 1	Alexa Fluor 647	VF1-17076	γHV68
Murid Herpesvirus TMER Type 4	Alexa Fluor 488	VF4-17586	γHV68
Mouse β-Actin Type 1	Alexa Fluor 647	VB1-10350	*Mus musculus*
Mouse β-Actin Type 4	Alexa Fluor 488	VB4-10432	*Mus musculus*
Mouse β-Actin Type 6	Alexa Fluor 750	VB6-12823	*Mus musculus*
HHV4 EBER 1-2 Type 1	Alexa Fluor 647	VF1-12409	EBV
HHV8 ORF73 Type 1	Alexa Fluor 647	VF1-6000252	KSHV
HHV8 T1.1 Type 4	Alexa Fluor 488	VF4-6000059	KSHV
Human β-Actin Type 6	Alexa Fluor 750	VA6-10506	*Homo sapiens*
Human IL-6 Type 10	Alexa Fluor 568	VA10-13146	*Homo sapiens*

Table B: TaqMan PCR probe sets used in this study

Primer/Probe Name	Sequence (5'-3')	Annealing Temp. (C°)
γHV68 Forward Primer	GGC CCA AAT TCA ATT TGC CT	60 C°
γHV68 Reverse Primer	CCC TGG ACA ACT CCT CAA GC	60 C°
γHV68 Probe FAM/TAMRA	ACA AGC TGA CCA CCA GCG TCA ACA AC	60 C°
Host NFAT5 Forward Primer	CAT GAG CAC CAG TTC CTA CAA TGAT	60 C°
Host NFAT5 Reverse Primer	TGC TTT GGA TTT CGT TTT CGT GAT T	60 C°
Host NFAT5 Probe VIC/HBQ	ACG AGG TAC CTC AGT GTT	60 C°

Table C: qPCR primer sets used in this study

PCR primer	Sequence 5'-3'	Tm °C
HV68 tRNA5 sense	GCC AGG GTA GCT CAA TTG	52 °C
miR-M1-12 RLMRTPCR	AAG GGG TAG GAC TCC CAC	52 °C
Mouse bActin Sense	GCC ACC AGT TCG CCA TGG	56 °C
Mouse bActin Antisense Inner	CAG GGT CAG GAT ACC TCT CTT G	56 °C
ORF73 RT-PCR Reverse Primer	GAG CCC CCT ACA GAG CCC CC	59 °C
ORF73 RT-PCR Forward Primer	CAC CTT GCT CAC CGG CA	59 °C
Hu bActin SYBR green Forward	GAT GAG ATT GGC ATG GCT TT	60 °C
Hu bActin SYBR green Reverse	CAC CTT CAC CGT TCC AGT TT	60 °C
KSHV ORF50 Forward	TCC GGC GGA TAT ACC GTC AC	60 °C
KSHV ORF50 Reverse	GGT GCA GCT GGT ACA GTG TG	60 °C
